# Anxiety and depressive disorders in elderly with chronic dizziness of vestibular origin^☆^

**DOI:** 10.1016/j.bjorl.2015.04.015

**Published:** 2015-10-09

**Authors:** Érica Toledo Piza Peluso, Maria Inês Quintana, Fernando Freitas Ganança

**Affiliations:** a Professional Master's Program in Body Balance Rehabilitation and Social Inclusion, Universidade Anhanguera de São Paulo, São Paulo, SP, Brazil; b Department of Psychiatry and Medical Psychology, Universidade Federal de São Paulo (UNIFESP), São Paulo, SP, Brazil; c Department of Otolaryngology and Head and Neck Surgery, Universidade Federal de São Paulo (UNIFESP), São Paulo, SP, Brazil

**Keywords:** Elderly, Anxiety, Depression, Dizziness, Idoso, Ansiedade, Depressão, Tontura

## Abstract

**Introduction:**

Dizziness is one of the most prevalent symptoms in the elderly. Anxiety and depression are common in dizzy adult patients, but there is scarce information about comorbidity between vestibular disturbances and psychiatric disorders in the aged.

**Objective:**

To assess the prevalence of anxiety and depression disorders in elderly with chronic dizziness of vestibular origin.

**Methods:**

Transversal study that used the Brazilian version of the Composite International Diagnostic Interview 2.1 to assess anxiety and depressive disorders in elderly patients (≥60 years old) with chronic dizziness.

**Results:**

Most of the 44 patients included in the study were female (88.6%) with a mean age of 71 years (±7.5), 68.1% had experienced dizziness for 1 year or more. The most prevalent diagnosis was benign paroxysmal positional vertigo (52.3%). The prevalence of generalized anxiety disorder and specific phobias during life were 29.5% and 22.7%, respectively, and, in the last 12 months, 18.2% and 15.9%. There was no patient with panic disorder, agoraphobia or social phobia. The prevalence of depressive disorder during life was 45.4%, and, in the last 12 months, were 11.3%.

**Conclusion:**

Aged patients with chronic dizziness had high prevalence of some mental disorders.

## Resumo

**Introdução:**

A tontura é um dos sintomas clínicos mais prevalentes entre idosos. Sintomas ansiosos e depressivos são frequentes em pacientes adultos não idosos com tontura, porém há pouca informação sobre a comorbidade das doenças vestibulares e transtornos mentais em idosos.

**Objetivo:**

Avaliar a prevalência de transtornos ansiosos e depressivos em idosos com tontura crônica de origem vestibular.

**Método:**

Estudo de corte transversal que utilizou como instrumento a versão brasileira do *Composite International Diagnostic Interview* 2.1 (CIDI) para avaliar transtornos ansiosos e depressivos em idosos (≥ 60 anos) com tontura crônica.

**Resultados:**

Foram incluídos 44 pacientes, a maioria do gênero feminino (88,6%), idade média de 71 anos, 68,1% apresentava tontura há mais de um ano, e 52,3% apresentavam diagnóstico de vertigem posicional paroxística benigna. A prevalência de transtorno de ansiedade generalizada e fobias específicas na vida foi de 29,5% e 22,7%, respectivamente, e, nos últimos 12 meses, de 18,2% e 15,9% respectivamente. Não houve casos de transtorno do pânico, agorafobia e fobia social. A prevalência de transtorno depressivo na vida foi 45,4%, e, nos últimos 12 meses, foi 11,3%.

**Conclusão:**

Encontrou-se elevada prevalência de certos transtornos mentais em idosos com tontura crônica.

## Introduction

Dizziness is the main symptom of vestibular diseases and one of the most prevalent symptoms in the population, being found even more often among the elderly.^1^, ^2^ Several factors may contribute to the onset of dizziness or aggravate this condition in individuals in this age group, including the aging of systems related to bodily balance, presence of chronic diseases associated with continuous use of drugs and, sometimes, with polypharmacy, among others.^3^

Dizziness can be intense, leading to loss of balance and to falls; usually its onset is accompanied by auditory and neurovegetative symptoms. In addition, many patients suffer an intense emotional distress and symptoms of anxiety and depression.^4^, ^5^ The physical and psychological symptoms limit physical and social activities of patients, and may affect dramatically their quality of life.^6^ Patients presenting with associated psychological symptoms are those who are more likely to remain symptomatic and disabled, with the highest levels of disability.^4^, ^7^

Several studies have evaluated the emotional aspects of non-elderly adult patients with dizziness of vestibular origin, reporting high prevalence of symptoms of anxiety and depression.^4^, ^8^, ^9^, ^10^ On the other hand, the literature is scarce on the emotional aspects of elderly people with dizziness of vestibular origin.

Anxiety and depression can be evaluated as diagnostic categories or as a *continuum*, using symptom scales. These forms of assessment are known as categorical and dimensional, respectively.^11^

Studies that choose the dimensional approach evaluate symptoms of anxiety and depression through instruments that measure their intensity, as the Geriatric Depression Scale (GDS), the Beck Depression Inventory (BDI), the State-Trait Anxiety Inventory (STAI), the Hospital Anxiety and Depression Scale (HADS), and others.

Studies evaluating elderly patients with vestibular diseases by these instruments identified high prevalence of depressive and/or anxiety symptoms.^12^, ^13^, ^14^

Few studies use the categorical approach in patients with chronic dizziness or vestibular disease. This approach provides psychiatric diagnoses according to current psychiatric classifications, including the International Classification of Diseases, 10th edition (ICD-10), and the Diagnostic and Statistical Manual of the American Psychiatric Association, 4th edition (DSM-IV).

Persoons et al.^15^ used the PRIME-MD PHQ for tracking the presence of psychiatric (somatoform, anxiety and depressive) disorders in 268 patients with complaints of dizziness (with or without vestibular disorder) with mean age of 48.2 years (SD 12.9). The authors found a prevalence of 35.8% of anxious or depressive disorders, and panic disorder was the most prevalent. Patients with psychogenic dizziness had higher rates of psychiatric disorders.

Eckhardt-Henn et al.^16^ evaluated 189 consecutive patients aged 19–64 years with dizziness, using a structured interview to assess the prevalence of mental disorders; their results indicated that 68.2% had a psychiatric (somatoform, anxiety or depressive) disorder. Comorbidities among patients with vestibular disease and psychiatric disorder were observed in 16.0%; and psychiatric disorders without vestibular disease were observed in 52.1%. The most prevalent diagnosis was that of anxiety and phobic disorders (30.0% of the total sample), followed by depressive disorders (28.0% of the total sample).

The comorbidity of various organic vertigo syndromes and psychiatric disorders was assessed by Eckhardt-Henn et al.^17^ in a sample of 68 patients with mean age of 49 years (SD = 14 years), compared to a control group of 30 healthy people, using a structured psychiatric interview (SCID-I). High rates of psychiatric comorbidity were found in patients with Ménière's disease (57.0%) and vestibular migraine (65.0%), especially with anxiety and depressive disorders. Patients with benign paroxysmal positional vertigo (BPPV) and vestibular neuritis had rates similar to those of control group (20.0%).

We found no studies addressing the association of dizziness of vestibular origin and mental disorders focusing on the elderly population.

The prevalence of mental disorders in this age group is different from that in young adults. In addition, there are differences in terms of etiology, symptoms, evolution and management of these clinical conditions in relation to other age groups.^11^, ^18^

The aim of this study was to evaluate the prevalence of anxiety and depressive disorders in elderly patients with chronic dizziness of vestibular origin.

## Methods

### Study design: cross-sectional cohort

#### Study location and participants

Data collection was conducted from September 2012 to September 2013 in Laboratório de Pesquisas do Programa de Mestrado Profissional em Reabilitação do Equilíbrio Corporal e Inclusão Social of Universidade Anhanguera, São Paulo. Consecutive elderly patients (≥60 years) with complaints of dizziness for at least 3 months and with a diagnosis of vestibular disease confirmed through clinical assessment performed by an otolaryngologist, which included history, physical examination and vestibular examinations, were evaluated.

Patients who were performing vestibular rehabilitation, patients who failed to respond to the questionnaires due to conditions that could compromise the communication, and patients with memory complaints were excluded.

### Instruments

#### Composite International Diagnostic Interview, version 2.1 (CIDI 2.1)

Anxiety and depressive disorders were assessed using the Brazilian version of the Composite International Diagnostic Interview, version 2.1 (CIDI 2.1). This instrument is a standardized and fully structured interview with the aim to diagnosing and classifying mental disorders. CIDI was developed in 1980 by the World Health Organization in collaboration with the US Alcohol, Drug Abuse and Mental Health Administration (ADAMHA). This instrument can be applied in epidemiological studies, clinical trials and at research centers by trained lay interviewers.^19^ In Brazil, CIDI 2.1. was validated by Quintana et al.^19^ Its sensitivity and specificity for depressive disorders was 82.5% and 92.8%, respectively, and for phobic–anxiety disorders, 80.6% and 93.5%, respectively. CIDI consists of 14 sections, with 10 sections for diagnostic purposes. In the current study, we used D (Phobia, Anxiety and Panic) and E (Depressive Disorders) sections. The diagnoses were established according to the International Classification of Diseases, 10th edition (ICD-10), taking into account two periods: the patient's lifetime and his/her last 12 months. We evaluated the following anxiety disorders: generalized anxiety disorder (GAD), specific phobias, agoraphobia, social phobia and panic disorder. The depressive disorders evaluated were depressive episode (of mild, moderate or severe intensity) and recurrent depression.

### Questionnaire for socio-demographic and clinical data of patients

#### Statistical analysis

The results were analyzed descriptively. Absolute and relative frequencies of responses for categorical variables were presented, and mean and standard deviation for continuous variables were calculated.

#### Ethical aspects

The project was approved by the Ethics Committee of the Universidade Anhanguera, São Paulo (protocol 113/10). All patients included in the study read and signed the Informed and Free Consent Form. Patients who were identified with any of the evaluated disorders or with significant emotional distress at the time of the interview were advised and referred to specialist services.

## Results

The sample consisted of 44 patients aged 60–90 years with mean age of 71 years and standard deviation (SD) of 7.5 years. There was female predominance (88.6%) among patients. Schooling ranged from 1 to 15 years of study, with a mean of 5.7 (SD = 3.7) years of study. Eighteen patients (40.9%) were married and only one was in paid work; the others were retired.

As to otoneurologic data, a higher prevalence of vertigo (61.4%) was noted; among these patients, 45.5% suffered daily dizziness, and 68.1% of all patients reported dizziness for more than one year. The most prevalent otoneurologic diagnosis was BPPV (52.3%).

With respect to mental disorders, GAD states, specific phobias and depression were identified. During their life, there was a predominance of depressive disorder states, especially moderate or severe depressive episodes; on the other hand, in the last 12 months there was a higher prevalence of GAD. No patients were diagnosed with agoraphobia, social phobia and panic disorder.

The prevalence of mental disorders is shown in Table 1.

**Table 1:** Prevalence of anxiety and depressive disorders, according to ICD-10, in elderly patients with chronic dizziness of vestibular origin (*n* = 44).

Disorder	Lifetime (*n*)	Lifetime (%)	12 months (*n*)	12 months (%)
**GAD**^a^	13	29.5	8	18.2
**Specific phobias**	10	22.7	7	15.9
**Depression** (total)	20	45.5	5	11.4
*Episode*	15	34.1	3	6.8
Mild	–	–	–	–
Moderate	9	20.5	3	6.8
Severe	6	13.6		–
*Recurrent depression*	5	11.4	2	4.6

a Generalized anxiety disorder.

In the sample, 8 patients (18.2%) were performing some kind of treatment in the mental health area.

## Discussion

With regard to socio-demographic characteristics of the sample, our participants were predominantly female, which is in accordance with the highest prevalence of dizziness and vestibular diseases in this group.^2^, ^3^, ^20^ In relation to clinical and neurotological aspects of patients, BPPV stood out; this is the vestibular disease most commonly found in the elderly.^3^, ^21^ Most of the elderly in this sample had dizziness for over a year.

The elderly in this study showed anxiety disorders (GAD and specific phobias) and moderate-to-severe depressive disorders in their lifetime and in the last 12 months; some patients had recurrent depression. No patient was diagnosed with panic disorder, agoraphobia, and social phobia.

The lifetime prevalence was much higher than that in the last 12 months. One explanation for this difference is the life span of elderly patients, because in this age group the patient has a much higher cumulative risk.^22^ Furthermore, anxiety and depressive disorders are more prevalent in young adults *versus* elderly people, with onset concentration of these conditions until the fifth decade of life.^22^

Elderly patients in this study exhibited several factors that are mentioned in the literature as risk factors for development of anxiety and depression, including poor schooling, female gender, retirement, and presence of chronic disease.^18^, ^23^

Viana and Andrade,^22^ in their study with a probabilistic sample of 5037 adults living in the metropolitan region of São Paulo and using CIDI 2.1, found 8.9% of the elderly (≥65 years) with specific phobias, 4.5% with TAG and 11.8% with depressive disorders in their lifetime. The prevalence varied significantly according to gender, being higher in women (16.5%, 4.5% and 23%, for each of the disorders above mentioned, respectively).

When compared with the prevalence in the community aforementioned, it is observed that the prevalence of lifetime depressive and anxiety disorders in the current sample is much higher than that found in the community, even when the comparison takes into account only female patients.

This finding may suggest that there is increased susceptibility to the development of clinical pictures of chronic dizziness in individuals with history of depressive or anxiety disorders. However, it has not been possible to corroborate this hypothesis in the literature, since no studies assessing patients with mental disorders before and after the onset of dizziness were published.

Regarding the prevalence in the last 12 months in the current study, 18.2% of patients were diagnosed with GAD, 15.9% with specific phobias and 11.3% with depression.

In America, Byers et al.^24^, using CIDI on a probabilistic sample of 2575 community-based individuals aged ≥55, indicated that, in the last 12 months, 6.5% had specific phobias, 2.0% had GAD, and 4% had major depression. It can be seen that the prevalence of GAD, phobias and depression in our study were higher than those in the study by Byers et al.^24^

When compared to community-based studies of prevalence of psychiatric disorders using the same instrument as the present study, it can be observed that the prevalence of anxiety and depressive disorders in this study was much higher. However, it should be emphasized that the diagnoses in this study were based on the classification of ICD-10; on the other hand, other population studies used the classification of DSM IV, which can lead to differences in prevalence due to different diagnostic criteria used by these classifications.

The comparison of this study *versus* studies that assessed patients with dizziness should take into account two aspects. First, there are few studies evaluating psychiatric disorders in patients with dizziness, and not one of them assessed only elderly patients. Another aspect limiting the comparison is the use of different diagnostic tools, psychiatric classification systems (DSM-IV) and samples (for instance, patients with psychogenic dizziness).

The study by Persoons et al.^15^ evaluated patients with complaints of dizziness; 53.4% had vestibular disease, and 27.2% had psychogenic dizziness. In addition, the authors used an instrument for psychiatric disorder screening, which may explain the higher prevalence found in their sample. Another aspect to be taken into account is that patients older than 75 years had been excluded from the study; and the mean age of their patients was much lower than that in the current study.

In the study by Eckhardt-Henn et al.^16^ assessing the prevalence of psychiatric disorders in patients with complaints of dizziness, younger patients were included (up to 65 years old), and most had no vestibular disease, but exhibited dizziness of psychogenic origin. These differences between samples may have influenced the higher prevalence of psychiatric disorders found by the authors of that study (68.2%). Despite this difference, these authors found similar results to those in our study with respect to specific disorders: 30% of the total sample in Eckhardt-Henn et al. study^16^ had a recent diagnosis of anxiety and phobic disorders – a figure similar to that found in our study (34.1% in the last 12 months). On the other hand, the prevalence of depressive disorders was lower (11.3% in the past 12 months) than that in Eckhardt-Henn et al.’ study (28%).^16^

In another study by Eckhardt-Henn et al.^17^ including 68 patients with vestibular disease, these authors observed a higher prevalence of psychiatric disorders *versus* that in the current study. This discrepancy may be partly explained by differences in the samples. Eckhardt-Henn et al.^17^ sample excluded elderly people, and most had a diagnosis of vestibular migraine, Meniere's disease and vestibular neuritis. Patients with BPPV, representing more than half of our sample, showed the lowest percentage of comorbidity with psychiatric disorders (10.0% were diagnosed with anxiety disorder, and 10.0% had depression) – results compatible with the current study with respect to depression.

A second aspect to be addressed is the comparison of results of this study, which held a categorical approach, with other studies that conducted a dimensional evaluation (symptoms of anxiety and depression) in patients with vestibular disease. Symptoms of anxiety and depression are common in elderly patients with vestibular diseases, affecting about 50% of patients.^10^, ^13^, ^14^

The difference between the high prevalence of anxiety and/or depressive symptoms and the relatively lower prevalence of anxiety and depressive disorders is expected, since more stringent criteria are used for the diagnosis of these disorders.^11^ This discrepancy reflects differences between categorical and dimensional assessments, that are complementary and cannot be directly compared.

We must highlight some limitations of this study. This is a cross-sectional study; thus, it is not possible to claim the existence of a causal link among psychiatric disorders *versus* vestibular diseases in the elderly. Longitudinal studies with larger samples are needed, in order to obtain a better understanding of this relationship.

In spite of these limitations, this study indicates that there is significant comorbidity of vestibular diseases and emotional problems in the elderly with chronic dizziness, as well as the need for an interdisciplinary approach to these patients.

Psychological and/or psychiatric assessment is relevant to the identification of patients, in whom emotional problems must to be directly addressed, and can contribute to a better outcome for vestibular diseases and to improve the patient's quality of life.

## Conclusion

Elderly patients with chronic dizziness of vestibular origin have a high prevalence of generalized anxiety disorder, specific phobias and major depression in their lifetime and in the last 12 months. There were no patients with panic disorder, agoraphobia and social phobia.

## Conflicts of interest

The authors declare no conflicts of interest.
